# Sealed Gravitational Capillary Viscometry of Dimethyl Ether and Two Next-Generation Alternative Refrigerants

**DOI:** 10.6028/jres.117.014

**Published:** 2012-09-26

**Authors:** Dylan S. Cousins, Arno Laesecke

**Affiliations:** National Institute of Standards and Technology, Boulder, CO 80305-3337

**Keywords:** *ab initio* calculations, adjustment, calibration, dimethyl ether, fluoropropenes, measurements, R1234yf, R1234ze(E), refrigerants, sealed gravitational capillary viscometer, viscosity

## Abstract

The viscosities of dimethyl ether (DME, C_2_H_6_O) and of the fluorinated propene isomers 2,3,3,3-tetrafluoroprop-1-ene (R1234yf, C_3_H_2_F_4_) and trans-1,3,3,3-tetrafluoropropene (R1234ze(E)) were measured in a combined temperature range from 242 K to 350 K at saturated liquid conditions. The instrument was a sealed gravitational capillary viscometer developed at NIST for volatile liquids. Calibration and adjustment of the instrument constant were conducted with *n*-pentane. The repeatability of the measurements was found to be approximately 1.5 %, leading to a temperature-dependent estimated combined standard uncertainty of the experimental data between 5.7 % at 242 K for dimethyl ether and 2.6 % at 340 K for R1234yf. The measurements were supplemented by *ab initio* calculations of the molecular size, shape, and charge distributions of the measured compounds. The viscosity results for dimethyl ether were compared with literature data. One other data set measured with a sealed capillary viscometer and exceeding the present results by up to 7 % could be reconciled by applying the vapor buoyancy correction. Then, all data agreed within the estimated uncertainty of the present results. Viscosities for the fluorinated propene isomers deviate up to 4 % from values predicted with the NIST extended corresponding-states model. The viscosities of the two isomers do not scale with their dipole moments. While the measured viscosity of R1234ze(E) with the lower dipole moment is close to that of R134a, the refrigerant to be replaced, that of R1234yf with the higher dipole moment is up to 25 % lower. The viscosity of dimethyl ether is compared with those of water and methanol.

## 1. Introduction

Viscosity measurements of volatile compounds are rare because instruments for samples that need to be contained are not as widely available or as conveniently operated as the ubiquitous and well standardized open glass capillary viscometers for non-volatile liquids [1]. Consequently, wide data gaps exist for the viscosities of volatile pure compounds and mixtures of compounds that consist of up to four carbon atoms.

A number of attempts were made to apply the gravitational capillary viscometer method to volatile sample liquids. Edwards and Bonilla [2] appear to have been the first to construct a sealed viscometer with a coiled capillary of thick-walled glass for measurements of 1,3-butadiene above atmospheric pressure. This type of instrument was used in several other studies over the next 46 years. However, while the viscosity results of Edward and Bonilla were confirmed within their estimated uncertainty of 3 % to 5 %, the later measurement results for halogenated hydrocarbons of Mears *et al.* [3], Phillips and Murphy [4,5], and Shankland *et al.* [6,7] were burdened with systematic errors of up to 33 %, the origin of which was not identified before another decade had passed.

In their development of sealed viscometers of thick-walled glass, Kumagai and Takahashi [8] tried to reduce or eliminate the coil in favor of straight capillaries. However, their measurement results for eleven halogenated hydrocarbons deviated systematically up to 14 % from other literature data.

Han *et al.* [9] enclosed a straight glass capillary viscometer according to Ostwald in a stainless steel pressure vessel with windows through which the efflux of the test sample through the capillary could be observed and timed. Their measurement results for the viscosity of hydrofluorocarbon 1,1,1,2-tetrafluoroethane (R134a) as well as those obtained with this instrument by Sun *et al.* [10] for difluoromethane (R32) and pentafluoroethane (R125) showed systematic deviations from literature data.

The causes of the systematic deviations were identified in NIST studies. For the characterization of alternative refrigerants, Ripple [11] developed a compact sealed gravitational capillary viscometer of stainless steel with a coiled capillary and published measurement results for six halogenated methane and ethane derivatives [12]. Similar to the development of Kumagai and Takahashi [8], the coiled capillary of the NIST viscometer was subsequently replaced by a straight capillary. Ripple and Defibaugh [13] pointed out in the context of their viscosity measurements of 1,1-difluoroethane (R152a), 1,1,1-trifluoroethane (R143a), and pentafluoroethane (R125) that some of the literature data obtained in sealed gravitational capillary viscometers were analyzed without accounting for the vapor buoyancy that becomes significant in such instruments. They showed that correcting the originally deviating literature data for this effect leads to a remarkable consistency with their experimental results. The influence of the vapor buoyancy was investigated further by Laesecke *et al.* [14] with measurements of ammonia, R134a, and R32 in the same instrument. In addition, the effect of radial acceleration on flow in coiled capillaries was quantified and demonstrated to exceed even that of the vapor buoyancy at certain conditions. Accounting for the vapor buoyancy in sealed instruments and for the radial acceleration in those with helically coiled capillaries reconciled all originally deviating viscosity measurements in gravitational capillary viscometers with the results measured with other instruments. Even the strong deviations of the data of Phillips and Murphy [4,5] could be reconciled, which had been inexplicable for nearly three decades. The paper of Laesecke *et al.* of 1999 [14] prompted Kumagai and Yokoyama in 2000 [15] to publish corrections for the effect of vapor buoyancy on the viscosity data for eleven liquids that had been published in 1991 [8]. The corrections removed the systematic deviations between the data of 1991 and literature data.

Apart from these benchmark studies, the NIST sealed gravitational capillary viscometer has played the role of a reference instrument in various other ways. Viscosity measurements of fluoropropane isomers that were published in 1996 [16] and in 1998 [17] were confirmed in 2011 within their estimated experimental uncertainties by measurements of Meng *et al.* with a vibrating wire viscometer [18]. Viscosity measurements of ten binary and ternary alternative refrigerant mixtures at saturated liquid conditions revealed highly non-ideal composition dependences in those mixtures that consist of a polar and a nonpolar compound [19]. In 2004, measurements of the binary mixture of 50 % (mass/mass) difluoromethane (R32) and pentafluoroethane (R125) in this instrument demonstrated that electroviscous effects occur when such polar compounds are subjected to alternating electric fields in the torsional crystal viscometer [20].

In this work, the sealed gravitational capillary viscometer was employed for measurements of dimethyl ether (DME, C_2_H_6_O) and two fourth-generation alternative refrigerants that are candidates to replace refrigerant R134a. The replacements should be as non-depleting of the stratospheric ozone layer as R134a, but have in addition much shorter atmospheric lifetimes for reduced global warming potentials. These objectives are met by fluorinated propenes which are a new class of fluids for the refrigeration industry [21]. Thermodynamic properties of 2,3,3,3-tetrafluoroprop-1-ene (R1234yf, H_2_C=CHF-CF_3_) have been published by Richter *et al.* [22] and those of trans-1,3,3,3-tetrafluoropropene (R1234ze(E), FHC=CH_2_-CF_3_) by McLinden *et al.* [23]. Thermal conductivity measurements of both fluorinated propenes were published by Perkins and Huber [24].

DME is the first member of the homologous series of ethers. The properties of this fluid are of great interest [25], but its viscosity has been measured only a few times. Due to its normal boiling point of 248.37 K and its vapor pressure of 0.59 MPa at 298.15 K, DME is a potential working fluid in vapor compression refrigeration cycles. Its ASHRAE designation is E170. A growing interest in DME arises from its use as a clean burning fuel and as an oxygenating fuel additive. DME is promoted as a fuel by the International DME Association [http://www.aboutdme.org/].

Our experimental research is paralleled by quantum-chemical calculations of the molecular sizes, shapes, and charge distributions of the measured compounds. This perspective is useful in the planning of materials handling, of measurements, and in the interpretation of the experimental results. The viscometer and its operation are described in detail, and particular emphasis is placed on the uncertainty assessment, which has been extended by an additional contribution over earlier practices. The measured viscosities of the three compounds are compared with the sparsely available literature data and with values predicted with an extended corresponding-states model. They are also put in perspective relative to the viscosities of chemically similar compounds.

## 2. Materials and Methods

### 2.1 Materials

The DME sample used in this study was purchased from a commercial supplier with a stated molar purity of more than 99 %. The sample was used as received without further purification. After the viscosity measurements, the sample in the viscometer was analyzed by gas chromatography-mass spectrometry. No significant organic impurities were detected in either the gas or the liquid phases, but they contained small amounts of nitrogen, oxygen, and carbon dioxide.

The fluorinated propene samples used in this study were drawn from the same supply cylinders that were used in the thermal conductivity measurements of Perkins and Huber [24]. Analyses in this laboratory by gas chromatography combined with mass spectrometry and infrared spectrophotometry according to the protocols of Bruno and Svoronos [26,27] confirmed the supplier-quoted purity of R1234yf of greater than 99.96 % (mole/mole) and a purity of R1234ze(E) of greater than 99.993 % (mole/mole). The samples were degassed by four to eight freeze-evacuate-thaw cycles with liquid nitrogen and final pressures in the vapor space of 4 mPa (R1234yf) and 0.1 mPa (R1234ze(E)).

### 2.2 Molecular Characterization of Materials

One of the objectives of the present investigation is to relate thermophysical properties observed on the macroscale to molecular size, shape, and charge distribution on the nanoscale. Table 1 presents numerical data of these molecular characteristics for dimethyl ether, R134a, R1234ze(E), and R1234yf. They were obtained from *ab initio* calculations of the equilibrium geometries and charge distributions by use of the SPARTAN’10 software [28] with the hybrid density functional model and BY3LP+6-311G** polarization basis set for all compounds [29]. The molecular structures are shown in Figs. 1 and 2 in terms of their electron density isosurfaces at a fixed value of 0.002 electron · au^−3^, where 1 au is the Bohr radius of hydrogen. The surface defined by this value of the electron density contains approximately 99 % of a molecule [30,31]. The dotted regions of the surfaces signify areas that are inaccessible if a sphere of radius 0.1 nm, centered on a line normal to the isosurface and touching a point in the middle of the region, impinges on any other regions of the isosurface [32]. These inaccessible parts are indicative of the geometry-dependent void volume between molecules, which contributes predominantly to their packing density but is also relevant for their mutual friction, the molecular mechanism from which viscosity arises in liquids. The electrostatic potential is mapped onto the electron density isosurfaces with a color scale ranging from red (negative charge) to blue (positive charge). The color distribution indicates the nonuniform charge distribution across a molecule. Greater separation of colors reflects greater polarity of a molecule. This facilitates understanding of the contributions of electrostatic forces in addition to intermolecular interactions due to size and shape. These renderings convey significantly more realism in the perception of molecules than simple conventional models of rigid balls-and-sticks or fused hard spheres. They show molecules as smooth, soft, and deformable entities, and include the perspective of their electrostatic Coulomb forces, which are not visualized in the conventional models. The results of this work demonstrate that a more realistic view is important in the interpretation of otherwise counterintuitive experimental results.

Figure 1 shows the structure and charge distribution of the water, methanol, and dimethyl ether molecules to provide a background for the comparison of their viscosities in Sec. 4.4. If one of the two hydrogen atoms in water is replaced by a methyl group, the resulting molecule is methanol. If the hydrogen on the hydroxyl group in methanol is replaced by a methyl group, the resulting molecule is dimethyl ether. The molecular volume increases by approximately 0.02 (nm)^3^ with each additional methyl group. The geometry of the molecules is shown in Fig. 1 to scale, while the color scale for the electrostatic potential of all three molecules is that of dimethyl ether ranging from −171 kJ·mol^−1^ to 63 kJ·mol^−1^ [32]. In water and methanol the electrostatic potential varies on smaller ranges: in water from −44 kJ·mol^−1^ to 47 kJ·mol^−1^, and in methanol from −44 kJ·mol^−1^ to 46 kJ·mol^−1^. Each of the molecules is shown in two views. The upper row shows the view on the centers of negative partial charges at the oxygen molecule. The lower row shows views of the parts of the molecules with positive partial charges. The colorization indicates a greater charge separation in dimethyl ether than in water and methanol.

Figure 2 compares the structures and charge distributions of the fluorinated olefins to those of R134a, which they are to replace as working fluid in vapor compression cycles. The electrostatic potential scale for the three molecules is that of R1234yf from −63 kJ·mol^−1^ to 164 kJ·mol^−1^ [32]. R134a is more uniformly charged than the fluorinated olefins. R1234ze(E) and R1234yf are isomers that differ only in the position of one fluorine atom across the double bond. R1234yf has all the strongly electronegative fluorine atoms on one side of the double bond, which results in a large dipole moment (*µ* = 2.48 D). R1234ze(E) has a fluorine atom on the opposite side of the double bond. This reduces the polarity of the molecule, which is reflected in a lower dipole moment (*µ* = 1.27 D) than that of R1234yf. To our knowledge, this work presents the first calculations of the dipole moments of the two fluorinated propene isomers. The volumes of R1234yf and R1234ze(E) are similar and are 17 % greater than that of R134a. Though R1234ze(E) and R1234yf are larger molecules than R134a, all of the molecules have very similar polar surface areas because each has four fluorine atoms.

With all of the fluorine atoms positioned on one side of the C=C double bond in R1234yf, the bond is polarized and more reactive than in R1234ze(E). Polymerization of R1234yf was observed in the tests of Richter *et al.* [22] at 393.15 K and 12 MPa. However, when the pressure was lowered to 6 MPa, polymerization did not occur. The temperature and pressure ranges at which polymerization occurs are well beyond the conditions investigated in this work.

## 3. Viscometer

### 3.1 Sealed Gravitational Capillary Viscometer

The instrument used in this work was a sealed gravitational capillary viscometer that was developed at NIST [13]. Figure 3 shows the front view of the instrument. At the top is a sealing valve through which the evacuated viscometer is filled with the test sample. The upper reservoir is constructed of a stainless steel body with sapphire windows. Polytetrafluoroethylene O-rings seal the windows against the stainless steel body components. The instrument is rated to hold pressures up to 3 MPa. The highest vapor pressure in the present measurements was 1.9 MPa with R1234yf at a temperature of 340 K. The upper reservoir is connected to the lower reservoir by two stainless steel tubes. First is a straight, vertically oriented capillary with a nominal internal diameter of 0.236 mm and a length of 148 mm. This capillary is the flow impedance for the viscosity measurement. Second is a reflux tube with a nominal internal diameter of 3.9 mm. Figure 4 shows the viscometer mounted on a stand, which is placed in a thermostatting bath. A measurement is conducted by allowing the sample liquid to drain from the upper reservoir through the straight capillary into the lower reservoir while timing the descent of the liquid meniscus with a stopwatch. The stopwatch function of a cell phone was used for the timing in this work. The viscometer is attached to an axle that may be turned by using a crank above the liquid level of the bath to invert the viscometer. In this upside-down orientation, the test liquid flows back into the upper reservoir through the reflux tube. By turning the crank again, the viscometer is returned to the original position for another measurement. Thus, a one-way flow circuit is established in the instrument.

The temperature of the sample and instrument was maintained within ±10 mK by immersing the entire viscometer in a 75 L bath that is insulated by a 7.5 cm thick polystyrene foam enclosure. The water + ethylene glycol bath liquid was continuously stirred by a mechanical impeller. A precision temperature controller was used to maintain the temperature of the bath liquid by heating with electric coils. An external circulator provided cooling to the bath by pumping cooling liquid through four loops of corrugated stainless steel tubing underneath the impeller. The cooling liquid temperature was set to 2 K below the target measurement temperature. The temperature of the bath and the viscometer was monitored with an ITS-90 calibrated platinum resistance thermometer (PRT) placed next to the viscometer in the bath. The accuracy of the PRT is stated to be ±10 mK. An estimated overall uncertainty in the temperature measurement of ±20 mK accounts for the difference in temperature between the viscometer and the location of the PRT in the bath. The temperature was allowed to equilibrate for a minimum of 10 minutes before the first measurement run was conducted.

Wedlake *et al.* [33] developed the general working equation for gravitational viscometers, which is applicable to both sealed and open instruments. The viscosity *η* is given by

(1) $$\eta =c_{1}(\rho_{\text{SL}}-\rho_{\text{SV}})\Delta t-\frac{c_{2}\rho_{\text{SL}}}{\Delta t}+c_{3}\sigma ,$$

where *c*_1_, *c*_2_, and *c*_3_ are calibration constants, *ρ*_SL_ and *ρ*_SV_ are the densities of the saturated liquid and saturated vapor, respectively, and *σ* is the surface tension between the two phases. The first term in Eq. (1) is the Hagen-Poiseuille term, which describes the flow of the liquid through the capillary. The buoyancy of the vapor column above the liquid influences the effective driving head and therefore, the difference between the densities of the liquid and the vapor must be used in the first term. The vapor density may be neglected for open instruments when non-volatile compounds are measured, because the density of air may be considered negligible. In sealed instruments, this simplification must be carefully examined to avoid measurement errors that can easily reach ten percent or more. The second term in Eq. (1) is the Hagenbach-Couette correction for the kinetic energy dissipation in the liquid at the inlet and outlet of the capillary. The third term is a correction for the effects of surface tension at the walls of the capillary. Densities of saturated liquid and vapor phases of the compounds involved in this work were obtained from the correlations of Lemmon and Span for toluene [34], Span and Wagner for *n*-pentane [35], Wu *et al.* for dimethyl ether [36], Tillner-Roth and Baehr for R134a [37], McLinden *et al.* for R1234ze(E) [23], and Richter *et al.* for R1234yf [22]. Equation (1) relies on a known bulb volume *V* and the measured efflux time interval ∆*t* to calculate a volumetric flow rate of the liquid through a capillary. The volume *V* is lumped into the calibration constant *c*_1_.

The present instrument uses a modified version of Eq. (1). Measurements conducted at elevated pressures require that the volume of the instrument be kept small. Therefore, it is impractical to measure an efflux time over a known volume with the present instrument. Instead of measuring an efflux time interval, the present method determines a rate of descent $\dot{h}$ of the liquid meniscus in the upper reservoir and uses a known cross-sectional area to determine a volumetric flow rate. The viscosity *η* may then be evaluated from

(2) $$\eta =\frac{C_{1}}{\dot{h}}(\rho_{\text{SL}}-\rho_{\text{SV}})-C_{2}\rho_{\text{SL}}\dot{h},$$

where *C*_1_ and *C*_2_ are calibration constants. The first term is analogous to the Hagen-Poiseuille term in Eq. (1). The cross-sectional area *A* of the upper reservoir is lumped into the calibration constant

(3) $$C_{1}=\frac{\pi d^{4}gh}{128LA},$$

where *d* is the inner diameter of the capillary, *g* = (9.79601148 ±0.0000023) m·s^−2^ is the gravitational acceleration at Boulder, Colorado as determined at NIST in 1973, *h* is the driving pressure head, and *L* is the length of the capillary. The upper reservoir has a nominal width of 9.4 mm and a nominal depth of 12.7 mm, yielding a cross sectional area of 120 mm^2^, which is similar to that of Type 1 Ubbelohde glass viscometers with reservoirs of circular cross section. The second term in Eq. (2) is also a correction for the dissipation of kinetic energy in the liquid at the inlet and outlet of the capillary. The constant *C*_2_ in Eq. (2) will differ in dimension by a factor of length from the analogous constant in Eq. (1). The third term in Eq. (1) has no analog in Eq. (2). Instead, surface tension effects are lumped into the calibration constants *C*_1_ and *C*_2_ and are considered in the uncertainty assessment in Sec. 3.2 below.

A volatile material with well characterized viscosity is preferred to adjust the calibration constant *C*_1_ of this viscometer [14]. All certified viscosity reference standards are non-volatile liquids at ambient conditions. Therefore, *n*-pentane was used in this work because this is a liquid with which the vapor buoyancy should be taken into account during the calibration near room temperature. The sample was from a commercial source with a stated molar purity greater than 99 %. Analysis of the sample by gas chromatography and mass spectrometry showed the actual mole fraction of *n*-pentane to be 97.72 % with 1.6 % 2-methylbutane as a major impurity and the balance consisting of methylcyclobutane and cyclopentane. For the viscometer calibration, the sample was degassed by four freeze-evacuate-thaw cycles with liquid nitrogen, each time with a final pressure of 0.1 mPa in the vapor space over the frozen sample. Measurements of *n*-pentane were conducted in the sealed viscometer at 293.15 K, 298.15 K, and 313.15 K with four measurements at each temperature. Reference viscosity data were established by critical evaluation of the sources listed in the compilations of Stephan and Lucas [38], Liley *et al.* [39], and Lan-dolt-Börnstein [40] as well as the DIPPR database [41] and the NIST Thermodynamics Research Center [42]. The calibration constant and its standard deviation were determined to be

(4) $$C_{1}=\left(6.386\pm 0.015\right)\times 10^{12}m^{3}\cdot s^{-2}.$$

This value is 2.7 % higher and 0.45 % lower than previous determinations with toluene by Laesecke *et al.* [14] and by Ripple and Defibaugh [13], respectively.

The calibration constant *C*_2_ of the kinetic energy correction term is implemented according to Cannon *et al.* [43]

(5) C2=0.037ReA8πL,

where the Reynolds number is approximated by

(6) Re=4AπdρSL(ρSL−ρSV)h˙2C1.

In the present measurements the Reynolds number varied between 47 and 193 for dimethyl ether, between 50 and 270 for R1234ze(E), and between 77 and 337 for R1234yf.

Figure 5 illustrates the shape of the liquid meniscus in the upper reservoir. The test liquid will wet the sapphire windows and stainless steel walls with different contact angles. The rise in the corners of the reservoir influences the effective driving head of the liquid. Therefore, surface tension effects must be accounted for in the uncertainty analysis. Figure 6 shows the surface tension and the so called *Laplace ratio σ*(*ρ*_SL_ - *ρ*_SV_)^−1^ as a function of temperature for the calibration liquids and refrigerants. The data were obtained from Jasper for toluene [44], Somayajulu for *n*-pentane [45], Soares *et al.* for DME [46], Higashi *et al.* for R134a [47], Tanaka and Higashi for R1234yf and for R1234ze(E) [48]. The surface tensions and *Laplace ratios* of the fluorinated refrigerants are all similar, while dimethyl ether has higher values for each of these properties. Among the refrigerants, R1234yf has the lowest value for each of these properties, while R1234ze(E) has the highest. The calibration liquids have significantly higher values for each of the properties. The surface tension and *Laplace ratio* of *n*-pentane are closer to those of the alternative refrigerants than the respective properties of toluene, which reduces the uncertainty of the measurements due to differences in surface tension between the calibration standard and the test samples.

Measurements were performed by timing the descent of the liquid meniscus as seen through the window of the upper reservoir. A cathetometer was used to observe the liquid meniscus through an opening in the polystyrene insulation of the bath. At temperatures below 273 K, a viewing glass was placed by suction with a vacuum pump to the glass window of the thermostatting bath to prevent ice formation from obscuring the view of the liquid meniscus. The visibility of the upper reservoir was improved by a light shined through the bath from the opposite side of the cathetometer. The position of the meniscus was recorded as a function of time. The measured height interval was determined based on the viscosity of the liquid. For measurements of low viscosity at the upper end of the temperature range, longer height intervals (0.5 mm) were needed because the liquid passed through the reservoir quickly. For measurements of high viscosity at the low end of the temperature range, short height intervals (0.25 mm) were used so that the measurements would not take an excessive amount of time. Ten height positions of the meniscus were recorded over the course of each measurement run. Four measurements were conducted at each temperature. Measurements were started once the liquid meniscus had passed the rounded corners at the top of the upper reservoir, so that the effect of surface tension was constant throughout the measurement and constant cross-sectional area was ensured. The height of the upper reservoir is 7.9 mm between the rounded corners. Thus, the effective measuring range between the rounded corners is approximately 6 mm, depending on the surface tension of the liquid.

Measured meniscus heights as a function of time *h* = *h*(*t*) were observed to be linear and therefore, fall rates $\dot{h}$ were determined by fitting straight lines to the (*h*,*t*)-data. Figure 7 shows the data points of four successive runs of R1234ze(E) at 246.7 K and those of dimethyl ether at 350 K. These measurements represent the highest and lowest viscosities observed in this work. The average standard deviation of the time between each measured step is 0.61 s for R1234ze(E) at 246.7 K and 0.65 s for dimethyl ether at 350 K. While these values for the standard deviations between each step are quite close, the standard deviation between time steps in consecutive runs at the lower temperatures were generally greater because it is more difficult to measure the passage of the meniscus with the cathetometer when the meniscus moves slowly. This will be detailed in the next section.

### 3.2 Uncertainty Analysis

The contributions to the uncertainty of the present measurements are compiled in Table 2. Type A uncertainty includes the uncertainty in the determination of the rate of descent of the liquid meniscus and the uncertainties of the liquid and vapor densities. Type A uncertainty was calculated as described in NIST Technical Note 1297 [49]. For a measurand

(7) $$y=y\left(x_{1,}x_{2},\ldots ,x_{i}\right),$$

the combined standard uncertainty is given by

(8) $$u_{c}(y)=\sqrt{\sum_{i=1}^{N}{\left[\frac{\partial y}{\partial x_{i}}\cdot u(x_{i})\right]}^{2}},$$

where *N* is the number of influence quantities. The influence quantities are the rate of descent of the meniscus, $\dot{h}$, and the liquid and vapor densities, *ρ*_SL_ and *ρ*_SV_. The uncertainty in the fall time was estimated by assuming a position assignment uncertainty of ±0.1 mm at low temperatures and of ±0.05 mm at high temperatures. The position assignment occurs when the observer sees the liquid meniscus at the level indicated by the cathetometer and records the elapsed time with the stopwatch. Data points at the beginning and end of each run were assigned this uncertainty. The slopes between the lower start and higher finish time and the slope between the higher start and lower finish time were calculated. These slopes represent the uncertainty boundaries in the descent rate of the liquid meniscus through the upper reservoir. The uncertainties of the liquid densities are 0.1 % for all samples and the uncertainties of the vapor densities are 0.5 % for all samples.

The Type A uncertainties for the instrument in the present work were found to be 2.4 % at high temperatures to 5.2 % at low temperatures for dimethyl ether, 2.0 % at high temperatures to 4.9 % at low temperatures for R1234ze(E), and 2.0 % at high temperatures to 5.0 % at low temperatures for R1234yf. These values are higher than previous estimates because the position assignment uncertainty was added to the uncertainty budget in this work.

Type B uncertainty contributions are also listed in Table 2. Uncertainty due to the determination of the kinetic energy correction was calculated using the method outlined by Bauer and Meerlender [50], and 100 % of the kinetic energy correction was used as the uncertainty. The calibration constant determined from *n*-pentane in the present work is more likely to yield accurate results than the previous calibration constants determined from toluene due to the similarity of the vapor pressure and surface tension of *n*-pentane with that of the test samples. Therefore, an uncertainty contribution of 0.5 % has been applied for the calibration with *n*-pentane, based on previous work.

The uncertainty in the measured viscosity arising from surface tension can be described in three separate contributions: the difference in the surface tension of the calibration liquid and the test sample, the effective driving pressure head, and wall drainage films and corner rise in the upper reservoir. The uncertainty due to the difference in surface tension between *n*-pentane and the test samples is ±0.1 %, as Bauer and Meerlender [50] quote that this uncertainty is usually less than 0.1 %. Wedlake *et al.* [33] provide an estimate for the pressure head variations due to the effects of surface tension. A previous correlation of their tabulated data [14] was employed here

(9) $$\frac{h}{h_{\sigma }}=0.1571{\left(\frac{\sigma }{\rho_{\text{SL}}-\rho_{\text{SV}}}\right)}^{0.4208},$$

to calculate the relative reduction in the effective pressure head (*h*/*h_σ_*) in percent as a function of the *Laplace ratio σ*(*ρ*_SL_ - *ρ*_SV_)^−1^ in units of 10^−6^ N·m^2^·kg^−1^. This contributes up to 0.6 % at low temperatures for dimethyl ether. Wall drainage films and corner rise slow the emptying of the upper reservoir by the liquid. The greater the surface tension of the liquid, the more pronounced the effect will be. Therefore, dimethyl ether is assigned an uncertainty contribution of 2.0 %, R1234ze(E) of 1.5 %, and R1234yf of 1.0 %.

Uncertainty in the measured viscosity due to the uncertainty of the temperature measurement of 0.02 K was found to contribute at most 0.03 % to the uncertainty budget. The Type B uncertainty contributions are added in quadrature and listed in Table 2. The combined standard uncertainties are calculated by adding the Type A and Type B uncertainties in quadrature. They range from 5.7 % for dimethyl ether at 242.5 K to 2.6 % for R1234yf at 340 K.

## 4. Results and Discussion

The averaged measurement results are presented in Tables 3 to 5. Graphs of the data vs. temperature are presented in Figs. 8–13. Values calculated with the correlations or the extended corresponding-states (ECS) model [51] implemented in NIST’s computer program REFPROP Version 9.0 [52] provided benchmarks for comparisons of the present results. Comparisons with literature data were also made. The following sections discuss the comparisons for each compound individually. The effects of molecular size, shape, and charge distribution are examined by comparing the viscosities of the measured compounds with those of compounds of related chemical structures.

### 4.1 Dimethyl Ether

The experimental viscosity data for dimethyl ether are listed in Table 3. Each data point represents the average of all four runs at a given temperature. Figure 8 shows the experimental viscosity data for saturated liquid dimethyl ether of the present work and literature data of Sivebaek *et al.* [53], Wu *et al.* [54], and Meng *et al.* [55]. Sivebaek *et al.* enclosed a Cannon-Fenske glass capillary viscometer in a pressure vessel. Besides measuring many binary blends of dimethyl ether with biodiesel-related compounds, they reported one viscosity measurement for saturated liquid dimethyl ether at 298.15 K. To our knowledge, this was the first published viscosity measurement of saturated liquid dimethyl ether. The second series of such measurements was published by Wu *et al.* [54]. They adopted the sealed gravitational capillary viscometer design of Han *et al.* [9] and calibrated their instrument with ethanol and water. 24 measured viscosities with an estimated uncertainty of 3 % were reported for saturated liquid dimethyl ether at temperatures from 227.22 K to 342.45 K. Most recently, Meng *et al.* [55] published results of viscosity measurements of dimethyl ether with a vibrating-wire viscometer relative to the viscosity of toluene at 298.15 K and 0.1 MPa. The data include values for saturated liquid dimethyl ether from 243.15 K to 373.13 K and data for the compressed liquid in the temperature range from 253.15 K to 373.13 K with pressures between 5 MPa and 30 MPa. The combined expanded uncertainty of the viscosity data was quoted as 2 %.

Meng *et al.* reported also a wide-ranging correlation of the viscosity of dimethyl ether from the dilute gas to the compressed liquid. This is used in Fig. 9 as the baseline for a comparison of the available viscosity data for saturated liquid dimethyl ether as a function of temperature. The data of Meng *et al.* [55] deviate from the correlation in a sinusoidal pattern between −0.98 % at 348.13 K and 1.8 % at 268.14 K. The data reported in this work show a similar sinusoidal pattern of deviations from the correlation of Meng *et al.*, with systematic offsets to lower viscosities between 2 % and 3%. Thus, the two data sets agree within the estimated uncertainty of the present measurements, and are consistent in their viscosity-temperature dependence. The sinusoidal deviation pattern may arise from an overfit with polynomial terms in the correlation of Meng *et al.*

The data point of Sivebaek *et al.* [53] is 4.9 % lower than the correlation of Meng *et al.* The highest deviations are seen in Fig. 9 for the data of Wu *et al.* [54]. They increase systematically with temperature from −5.3 % at 227.22 K to 4.2 % at 345.45 K. This deviation pattern is typical for data from a sealed gravitational capillary viscometer when the vapor buoyancy correction is not applied. Wu *et al.* [54] considered the vapor buoyancy correction in their work but did not apply it to the measurements of dimethyl ether, due to a lack of reliable density data for the saturated vapor at that time. This gap has since been closed with the reference equation of state for the thermodynamic properties of dimethyl ether that was published in 2011 [36]. With densities calculated from this equation of state, the vapor buoyancy correction was applied to the viscosity data of Wu *et al.* [54] here by multiplying with the density ratio (*ρ*_SL_ – *ρ*_SV_)/*ρ*_SL_. The deviations of these corrected viscosities are also shown in Fig. 9. The correction contributes from −0.1 % at 227.22 K to −6.9 % at 342.45 K and brings the results of Wu *et al.* into agreement with the results of this work within their estimated uncertainty, particularly above 300 K. Below 300 K, the deviations of the corrected data of Wu *et al.* are systematically lower than the present results, and the greatest deviation is −3.7 % at 270 K. The noticeable change in the deviations of the data of Wu *et al.* [54] at 300 K suggests a systematic effect, the origin of which cannot be identified based on the available information.

In summary, the results of the present viscosity measurements of dimethyl ether support the vapor-buoyancy corrected data of Wu *et al.* [54] above 300 K that were measured with a different sealed gravitational capillary viscometer. In this temperature range, the two data sets are approximately 3 % to 4 % lower than the data of Meng *et al.* [55], measured in a vibrating wire viscometer. On the other hand, the present results are consistent with the temperature dependence of the data of Meng *et al.*, but are approximately 2 % lower. Further measurements with other techniques are needed to resolve this difference.

### 4.2 R1234yf (1,1,1,2-tetrafluoropropene)

The experimental viscosity data for R1234yf are listed in Table 4. Each data point represents the average value of all four runs at a given temperature. Figure 10 illustrates the viscosity data of the present work compared with those of Hulse *et al.* [56], those of Raabe and Maginn [57], and with values calculated with the extended corresponding-states model (ECS) model [51] implemented in NIST computer program REFPROP Version 9.0 [52]. The ECS model was adjusted to the data of Hulse *et al.* [56] because they were the only viscosity measurements of R1234yf in 2009. They were conducted with an oscillating piston viscometer. This instrument is designed for measurements of compressed liquids; phase equilibrium between saturated liquid and saturated vapor cannot be realized without modifications. Therefore, the viscosity of the compressed liquid R1234yf was measured along five isotherms from 257.45 K to 307.45 K with pressures from 0.33 MPa to 2.1 MPa. Raabe and Maginn [57] applied the AMBER force field [58] to fluoropropenes. The force field parameters were derived from a combination of *ab initio* calculations and adjustments to experimental vapor pressure and saturated vapor and liquid density data of R1234yf and R1234zf (3,3,3-trifluoro-1-propene). Shear viscosities were then obtained from equilibrium molecular dynamics simulations and Green-Kubo integration of the shear stress autocorrelation function [59].

Figure 11 shows the percent deviations of the available data from those calculated with the ECS model. The measurements of Hulse *et al.* [56] have a reported uncertainty of 0.02 mPa·s, which amounts to 7.7 % at the lowest temperature of 257.45 K and 14 % at the highest temperature of 307.45 K. The present measurements results agree with the data of Hulse *et al.* within ±2 %, except at their lowest temperature of 257.45 K, where they are between 3 % and 5.5 % higher. The deviations of the present data from those calculated with the ECS model indicate a systematically different temperature dependence, because they range from −4.3 % at 246.54 K to 4.2 % at 320.00 K. The results of Hulse *et al.* are consistent with this temperature dependence, except for the point at 257.45 K.

The molecular dynamics simulations of Raabe and Maginn [57] were performed at temperatures from 263.15 K to 310.0 K with pressures of 1 MPa, 1.5 MPa, and 2 MPa. The estimated uncertainties of the reported viscosities for R1234yf range between 4.0 % at 263.15 K and 1 MPa and 9.2 % at 283.15 K and 2 MPa. Compared to values calculated with the ECS model, the simulation results are between 2.2 % and 6.4 % lower. They are also lower than the results of this work, but are consistent with their temperature dependence. This agreement is remarkable, because no viscosity data were used in the force field development so that the viscosities from the molecular simulations are entirely predicted. The lower uncertainties and the extended temperature range of the present measurement results will improve the ECS model for the viscosity of R1234yf for more accurate data representation and more reliable predictions.

### 4.3 R1234ze(E) (trans-1,1,1,3-tetrafluoropropene)

The experimental viscosity data for R1234ze(E) are listed in Table 5. Each data point represents the average value of all four runs at a given temperature. There is only one prior data set for the viscosity of this compound in the literature. Figure 12 shows the measured viscosity data of the present work and those of Grebenkov *et al.* [60] in comparison with the viscosity of R1234ze(E) calculated with the ECS model [51] implemented in NIST computer program REFPROP Version 9.0 [52]. Grebenkov *et al.* measured the viscosity of R1234ze(E) in the compressed liquid phase with a viscometer where mercury provides the driving force for the flow of the test sample through the capillary. The range of the data includes five temperatures between 301.65 K and 368.85 K with pressures between 0.99 MPa and 6.08 MPa. The estimated uncertainty of the viscosity data is reported as 2.5 %. Deviations of the measured viscosities from the ECS model [51] are presented in Fig. 13. The data of Grebenkov *et al.* [60] are represented within ±0.5 %, because the shape factors in the model were adjusted to them. The measurement results of the present work are systematically lower than the model, with deviations between −1 % to −3 %. In the overlapping temperature range from 300 K to 340 K, the present results agree with those of Grebenkov *et al.* within their combined standard uncertainty, which decreases from 3.7 % to 2.7 %, respectively. In the temperature range below 300 K, the present results support the viscosity-temperature dependence in the ECS model, because they remain flat and do not show systematic deviations as the results for R1234yf in Fig. 11. The reasons for this agreement will be discussed in the next section.

### 4.4 Viscosity Comparisons

The experimental results for the saturated liquid viscosities of the two fluorinated propene isomers are shown in Fig. 14 and compared to the viscosity of R134a [14,51], which they are to replace. In terms of their viscosities, R1234ze(E) appears to be a most suitable replacement of R134a, because its viscosity agrees with that of the latter between −3.3 % and 4.4 % across the temperature range from 246.3 K to 340 K. Figure 14 shows that the temperature dependence of the viscosity of R1234ze(E) is slightly less pronounced than that of R134a. This may be interpreted as a result of the lower polarity of R1234ze(E) compared to that of R134a, as indicated by their dipole moments in Table 1 of 1.27 D and 2.058 D, respectively. The viscosities of polar liquids have more pronounced temperature dependences than those of nonpolar compounds because of electrostatic attractions due to the nonuniform charge distributions in such molecules [61]. These electrostatic attractions contribute to molecular interactions, especially at low temperatures, when the repulsive interactions are weak because of the lower kinetic energy of the molecules. With increasing temperature, the electrostatic attractions are increasingly overcome by the kinetic energy of the molecules.

In Fig. 14, the measured viscosity of R1234yf seems to contradict this view. While the *ab initio* calculations indicate a higher polarity of this molecule, resulting in a dipole moment of 2.48 D, which is 21 % higher than that of R134a (2.058 D, Table 1), the viscosity of R1234yf is significantly lower than that of R134a. At 246.5 K the difference is −26 %, reducing with increasing temperature to −15.6 % at 340 K. This lower viscosity indicates significantly weaker attractions and/or stronger repulsions in R1234yf than in its isomer R1234ze(E). This difference is even more remarkable, as it results from the position change of only one fluorine atom between the two compounds.

The key to reconcile these seemingly contradictory characteristics is the particular electronegativity of fluorine, which is the highest of all elements, and, in turn results in a very low polarizability of its electron cloud. Thus, fluorine atoms cause nonuniform charge distributions within molecules, but their tightly bound and stiff electron clouds may lead to repulsive interactions between molecules. As a result, fluorinated compounds run the gamut from very polar, such as hydrofluoric acid or difluoromethane, to very inert and stable compounds such as perfluorinated alkanes, sulfur hexafluoride, or polytetrafluoroethylene (PTFE). The four fluorine atoms of R1234yf are all positioned on one side of its C=C double bond. This creates a contiguous perfluorinated hard shell on the ethane side of the molecule that acts repulsively and exceeds the electrostatic attractions between these polar molecules, thus overall decreasing the viscosity of this compound. In R1234ze(E), one fluorine atom is “shifted” to the other side of the C=C double bond so that the repulsive centers are not contiguous. More dispersed opposite charges are seen in R1234ze(E) compared to R1234yf, where all of the positive and negative charges are at either end of the molecule (Fig. 2). This distributed polarity in R1234ze(E) allows for more intermolecular electrostatic attractions between H^…^F atoms, and this results in a higher viscosity than that of R1234yf. Earlier measurements with the present viscometer revealed such interactions in fluorinated propanes [16,17]. Computational studies of fluorinated ethanes also provided evidence for these interactions [62].

Now that reliable data are available, it is also helpful to put the viscosity of dimethyl ether in perspective with those of water and methanol, and to relate the differences to the molecular structures that are visualized in Fig. 1. The viscosities of the comparison liquids were calculated with the reference correlations by Huber *et al.* [63] and Xiang *et al.* [64], respectively. The viscosity-temperature graph for the saturated liquid of the three substances in Fig. 15 shows that strong hydrogen bonding in water causes it to have the highest viscosity and steepest temperature dependence of these compounds, although it is the smallest molecule of the three [65,66]. When one of the hydrogen atoms of water is replaced by a methyl group to give the structure of methanol, both the viscosity and its temperature dependence decrease, although the volume and surface area of the molecule increase. The decrease arises from the hydrophobic methyl group, which reduces the number of possible hydrogen bonds in the network of molecules in liquid methanol as compared to water [67]. Figure 15 shows that an even greater decrease of the viscosity and its temperature dependence occurs when the hydrogen in the OH-group of methanol is replaced by a methyl group to form dimethyl ether. Consequently, hydrogen bonds in liquid dimethyl ether are greatly reduced when compared to methanol. Nevertheless, electrostatic forces still contribute significantly to the interactions between dimethyl ether molecules.

## 5. Concluding Remarks

This work contributes in three areas: reliable viscosity data, improved metrology leading toward standardization, and molecular thermodynamics. The most important outcome are the measured viscosity data for the saturated liquid state of three compounds that have been studied only sparsely so far but are of increasing importance for greater efficiency and reduced environmental impact of our energy utilization.

The measurements were carried out with a sealed gravitational capillary viscometer with a proven track record. The development of this type of instrument is reviewed. The application of these instruments is marked by frequent omission of the vapor buoyancy correction in the data analysis. However, the vapor buoyancy can influence the flow of the sample liquid in such instruments significantly. One such case occurred in the scope of this work for the viscosity of dimethyl ether. Literature data measured with a sealed capillary viscometer and exceeding the present results by up to 7 % could be reconciled by applying the vapor buoyancy correction.

Omissions of this correction result from a lack of guidance for sealed capillary viscometers in reference books and standards. These address only open gravitational capillary viscometers, with one exception. However, while ASTM D4486 *Standard Test Method for Kinematic Viscosity of Volatile and Reactive Liquids* does cover sealed viscometers, it does not include the vapor buoyancy correction [68] either. It is hoped that this gap in standardization can be closed in the near future.

The measured viscosities of the two fluorinated propene isomers differ significantly, although the molecules differ only in the position of one fluorine atom relative to the double bond. Their viscosities do not scale with their dipole moments. Therefore, the dipole moment should not be the only factor when considering the polarity of a molecule. Rather, the charge distribution across an entire molecule should be taken into account as well as the polarizability of the electron cloud. This work is an example of an approach in which property measurements are combined with computational molecular science to achieve a better understanding of structure-property relationships.

## Figures and Tables

**Fig. 1:**
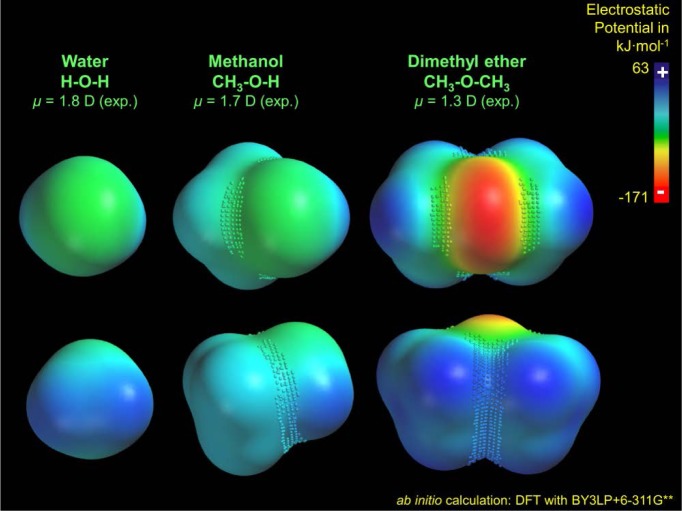
Molecular size, shape, and charge distribution of water, methanol, and dimethyl ether. The dots indicate areas that are inaccessible if a sphere of radius 0.1 nm centered on a line normal to the isosurface and touching a point in the middle of the region, impinges on any other regions of the isosurface [32].

**Fig. 2:**
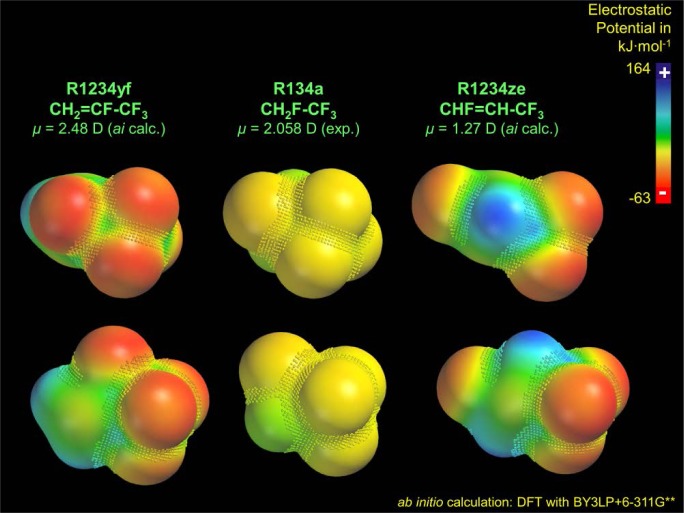
Molecular size, shape, and charge distribution of fourth-generation refrigerants compared to R134a. The dots indicate areas that are inaccessible if a sphere of radius 0.1 nm centered on a line normal to the isosurface and touching a point in the middle of the region, impinges on any other regions of the isosurface [32].

**Fig. 3:**
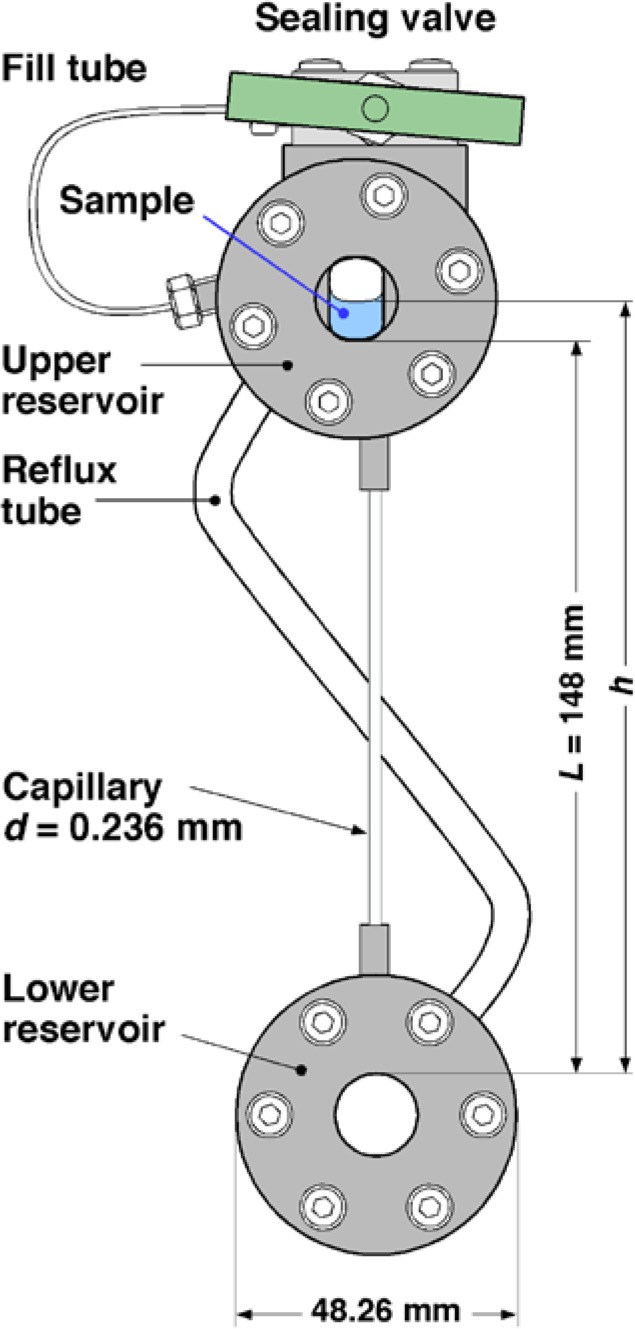
Sealed gravitational capillary viscometer with straight vertical capillary.

**Fig. 4:**
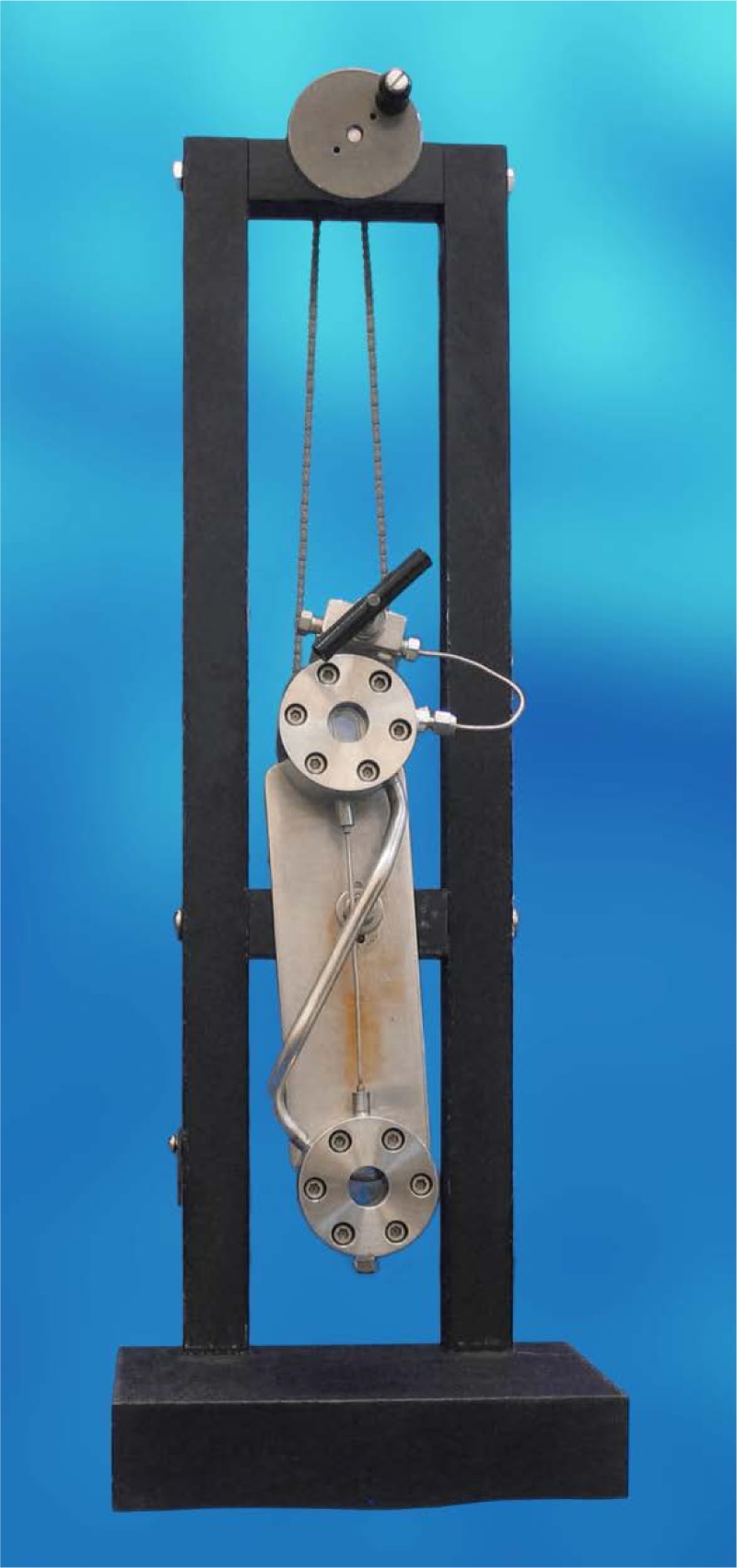
Viscometer mounted on stand with crank.

**Fig. 5:**
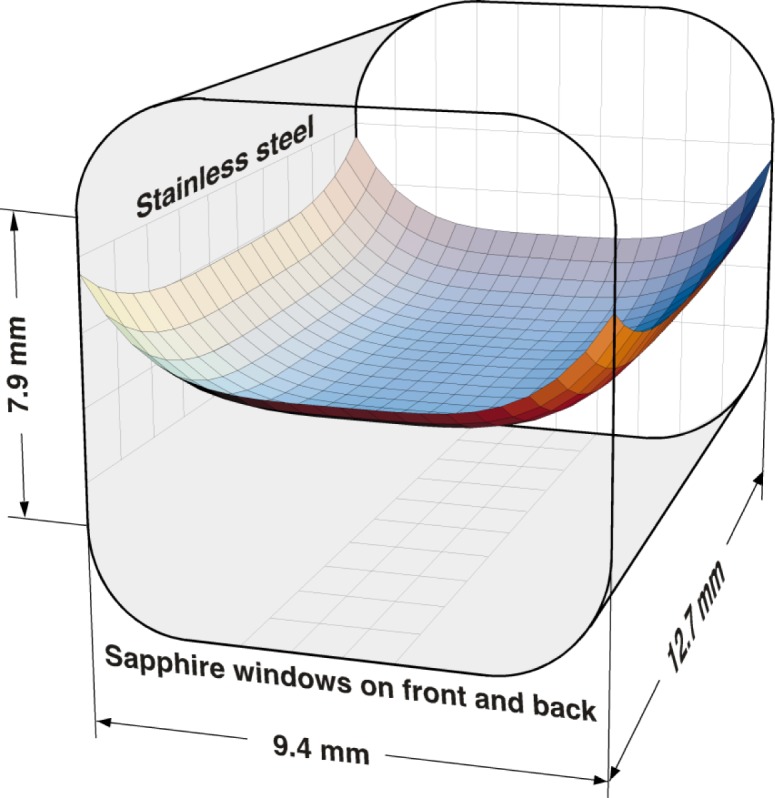
Illustration of liquid meniscus with corner rise in the upper reservoir of the viscometer.

**Fig. 6:**
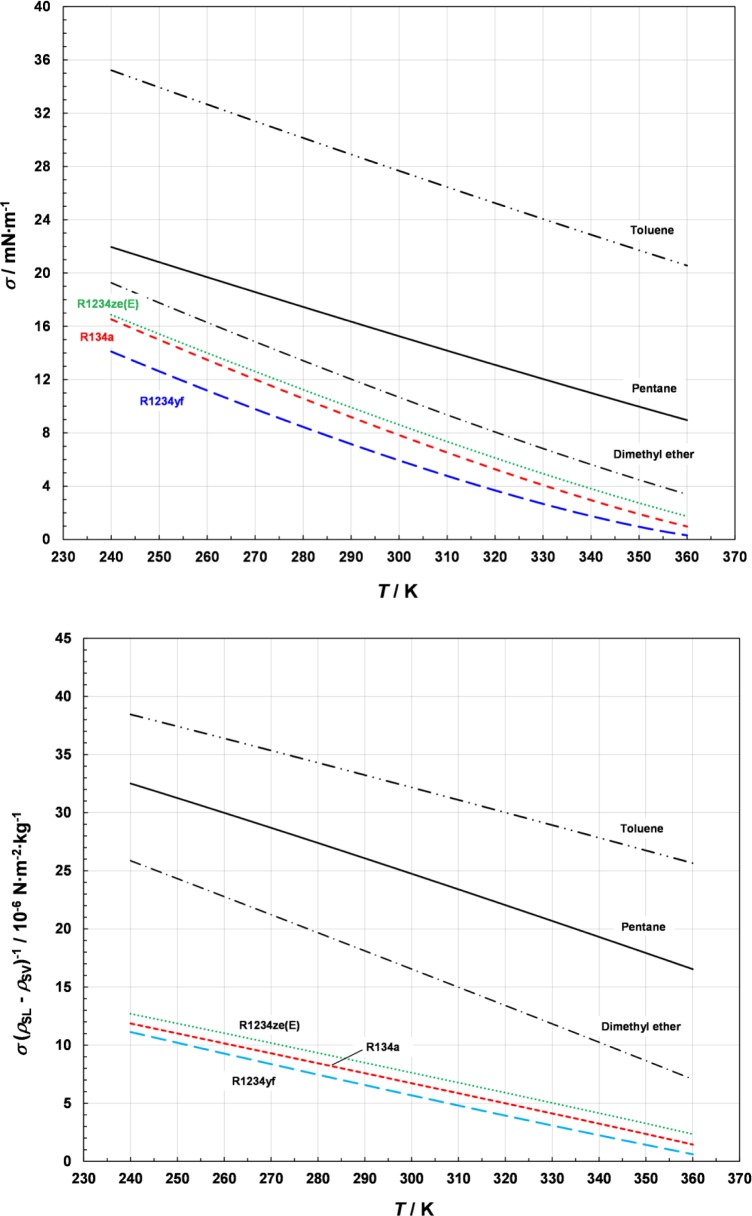
Vapor-liquid surface tension (top) and the Laplace ratio (bottom) of the measured fluids as a function of temperature.

**Fig. 7:**
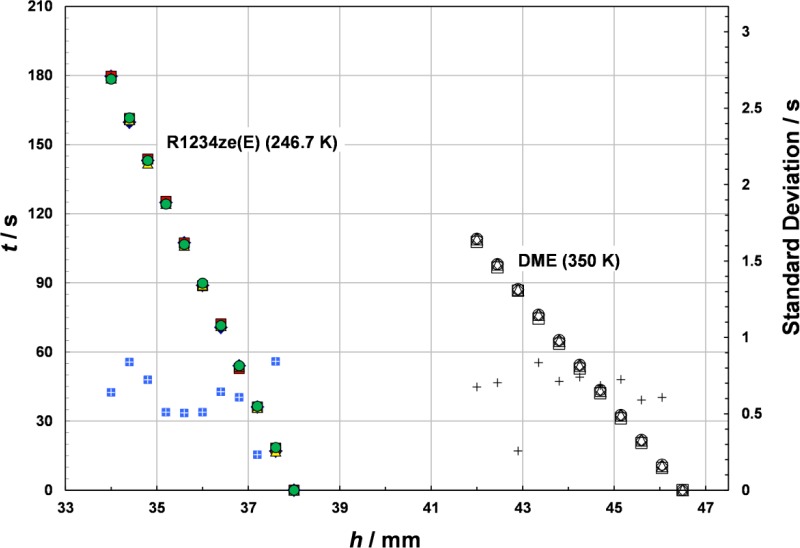
Four successive measurements of the passage of the meniscus of R1234ze(E) at 246.7 K and of dimethyl ether at 350 K. Cross symbols denote standard deviations of each measurement.

**Fig. 8:**
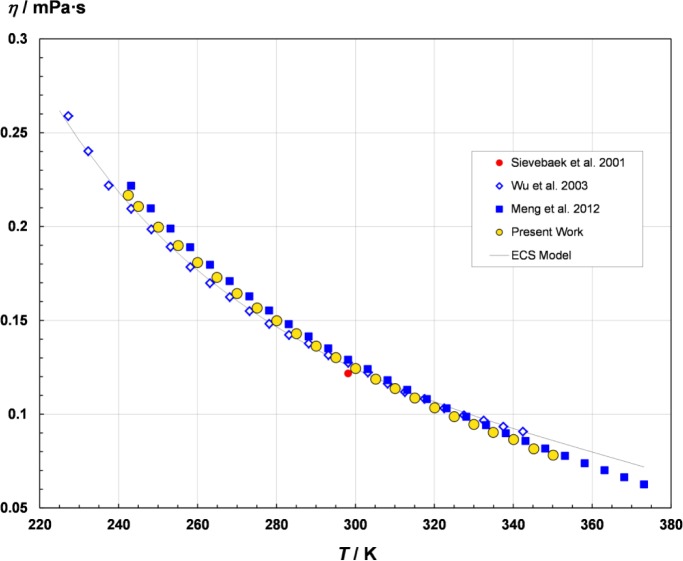
Measured dynamic viscosity of dimethyl ether as a function of temperature.

**Fig. 9:**
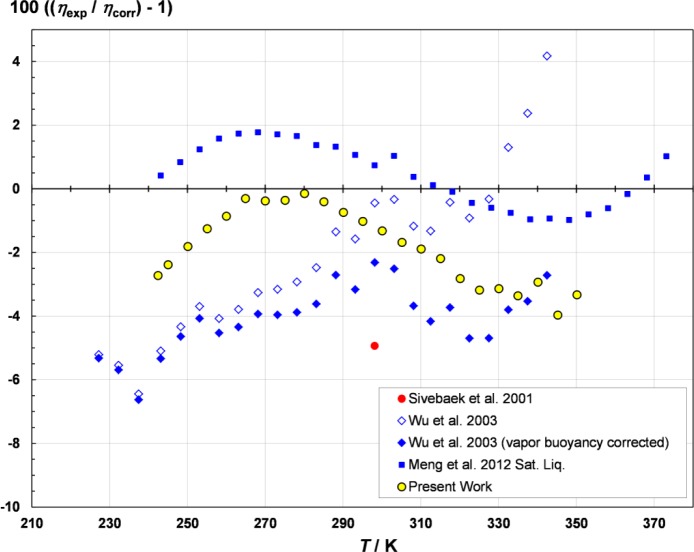
Percent deviations of measured dynamic viscosities of dimethyl ether from the wide-ranging correlation of Meng et al. [55] as a function of temperature.

**Fig. 10:**
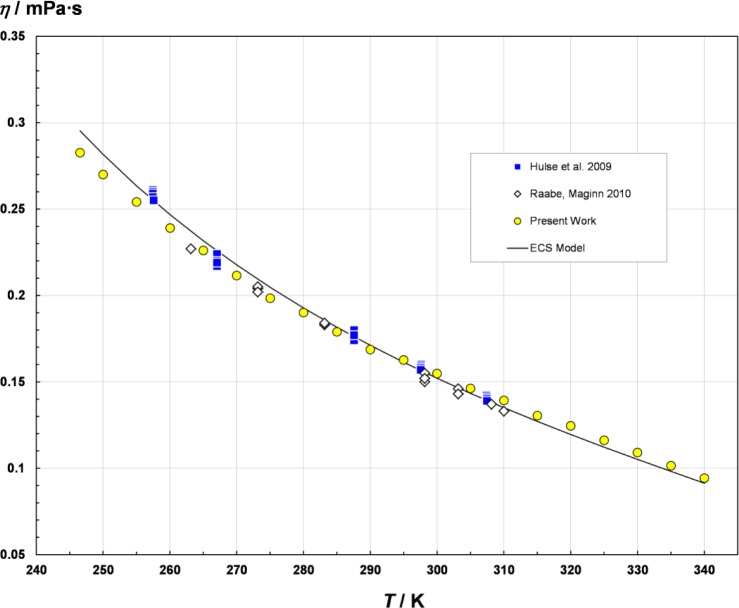
Measured dynamic viscosities of R1234yf and literature data as a function of temperature.

**Fig. 11:**
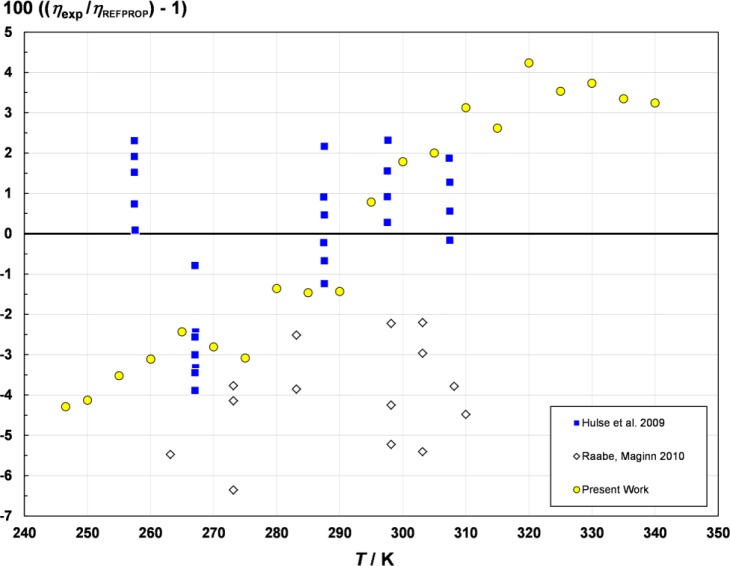
Percent deviations of measured dynamic viscosities of R1234yf from viscosities predicted with the ECS model implemented in NIST software REFPROP as a function of temperature.

**Fig. 12:**
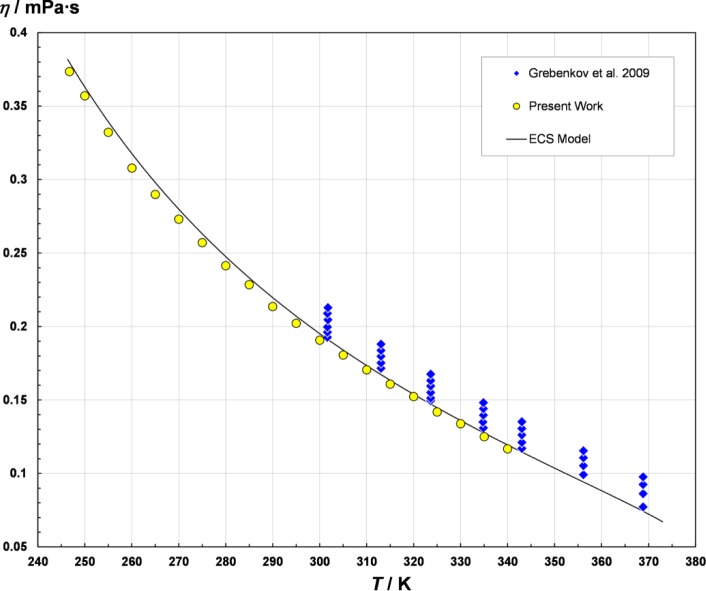
Measured dynamic viscosities of R1234ze(E) as a function of temperature.

**Fig. 13:**
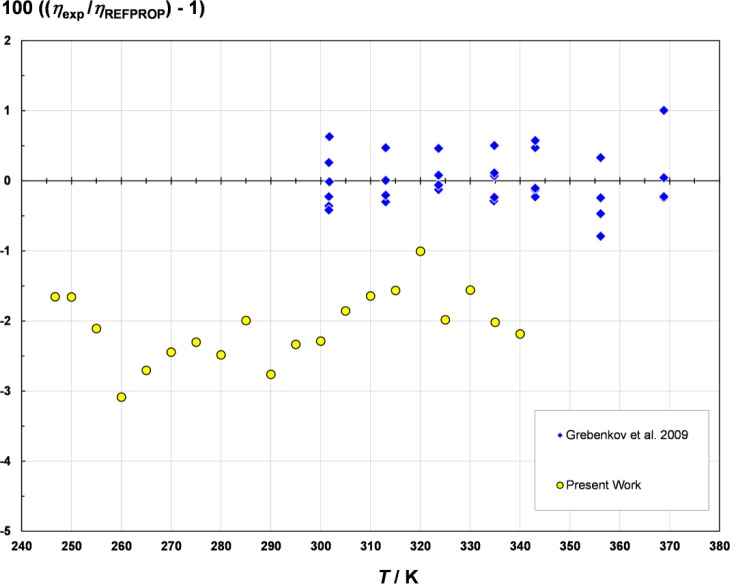
Percent deviations of measured dynamic viscosities of R1234ze(E) from viscosities predicted with the ECS model implemented in NIST software REFPROP as a function of temperature.

**Fig. 14:**
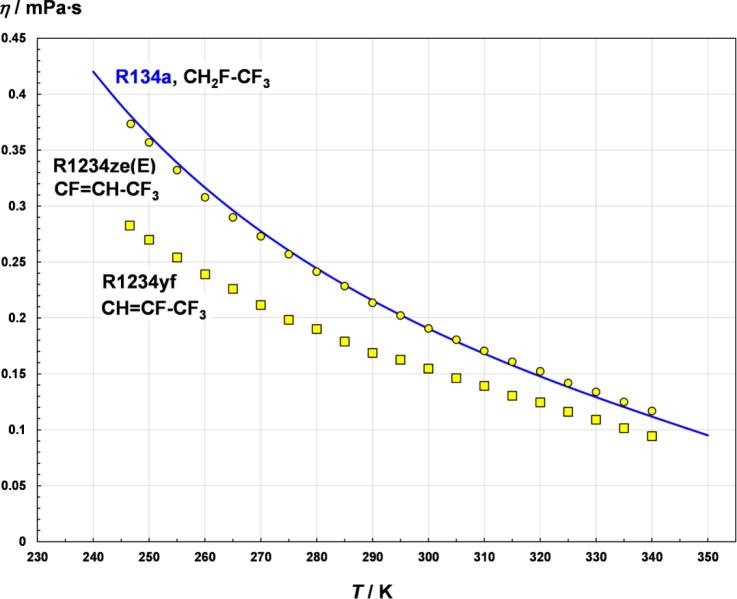
Dynamic viscosities of saturated liquid fluorocarbons R134a, R1234ze(E), and R1234yf as a function of temperature. The viscosities of R134a were calculated with the correlation of Huber et al. [51].

**Fig. 15:**
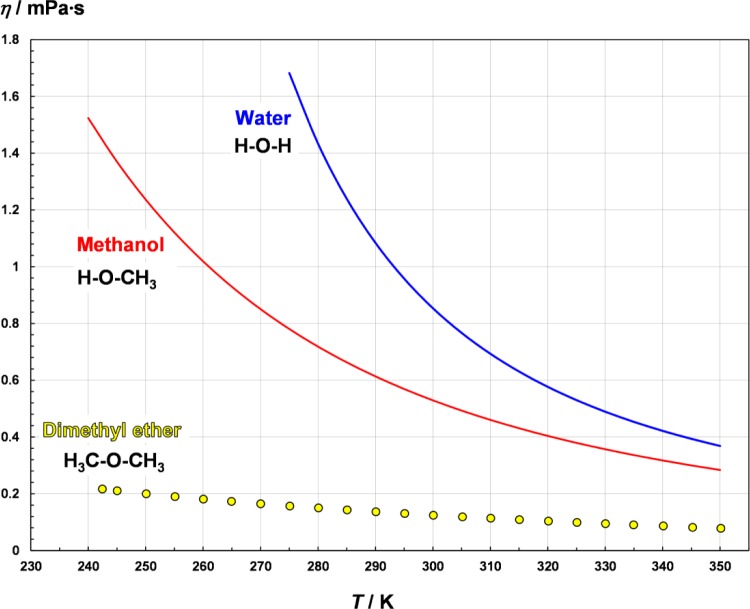
Viscosities of saturated liquid water, methanol, and dimethyl ether as a function of temperature. Data of water and methanol were calculated from the standard reference correlations of Huber et al. [63] and of Xiang et al. [64], respectively. Dimethyl ether data are from Table 3 of this work.

**Table 1:** Molecular characteristics of the investigated compounds

Property	Units	Dimethyl Ether	R134a	R1234ze(E)	R1234yf
Molecular Volume	(nm)^3^	0.06312	0.07262	0.08517	0.08528
Molecular Surface Area	(nm)^2^	0.8457	0.9528	1.081	1.074
Ovality	—	1.18	1.22	1.24	1.24
Accessible Surface Area	(nm)^2^	0.7250	0.6747	0.7935	0.7845
Polar Surface Area*)	(nm)^2^	0.0916	0.6789	0.6714	0.6698
Dipole Moment (calculated)	D	1.45	2.23	1.27	2.48
Dipole Moment (experimental)	D	1.30†)	2.058±0.01≠)	n/a	n/a

*) The polar surface area (PSA) is defined as the area due to oxygen and the hydrogens attached to oxygen. For the fluorinated compounds, the polar surface area is that of the fluorine atoms. For details see: P. Ertl, Polar Surface Area, in *Molecular Drug Properties: Measurement and Prediction* (Eds.: R. Mannhold, H. Kubinyi, G. Folkers), John Wiley & Sons, pp. 111–126 (2008).

†) R. D. J. Nelson, D. R. J. Lide, and A. A. Maryott, *Selected values of electric dipole moments for molecules in the gas phase*, National Bureau of Standards, Compilation, NSRDS-NBS 10, Washington, D. C., 49 pages (1967).

≠) C. W. Meyer and G. Morrison, *Dipole Moments of Seven Partially Halogenated Ethane Refrigerants*, J. Phys. Chem. **95** 9, 3860–3866 (1991).

**Table 2:** Uncertainty evaluation of the present measurements

	Dimethyl ether		R1234yf		R1234ze(E)	
Temperature range (K)	242.5	350	246.5	340	246.7	340
Type A Uncertainty (%)	5.2	2.4	5.0	2.0	4.9	2.0
	Type B Uncertainty Contributions (%)					
Kinetic energy correction	0.06	0.5	0.1	1.1	0.07	0.8
Calibration with *n*-pentane	0.5	0.5	0.5	0.5	0.5	0.5
Surface tension difference between calibration liquid and sample	0.1	0.1	0.1	0.1	0.1	0.1
Pressure head variation due to surface tension effects on curved interfaces	0.6	0.4	0.4	0.2	0.4	0.3
Wall drainage films and corner rise in upper reservoir	2.0	2.0	1.0	1.0	1.5	1.5
Density of saturated liquid	0.1	0.1	0.1	0.1	0.1	0.1
Density of saturated vapor	0.5	0.5	0.5	0.5	0.5	0.5
Uncertainty of temperature measurement (±0.02 K)	0.02	0.02	0.03	0.03	0.03	0.03
Total Type B Uncertainty (%)	2.2	2.2	1.3	1.7	1.7	1.9
**Combined Standard Uncertainty (%)**	**5.7**	**3.3**	**5.2**	**2.6**	**5.2**	**2.7**

**Table 3:** Averaged experimental viscosities for saturated liquid dimethyl ether

Temperature *T*	Pressure* *P*	Saturated Liquid Density* *ρ*_SL_	Saturated Vapor Density* *ρ*_SV_	Viscosity *η*	Combined Standard Uncertainty *u*(*η*)
K	MPa	kg·m^−3^	kg·m^−3^	mPa·s	%
242.46	0.07778	743.24	1.834	0.2166	5.7
245.04	0.08746	739.73	2.046	0.2106	5.6
250.11	0.1092	732.79	2.518	0.1996	5.4
255.11	0.1347	725.85	3.062	0.1898	5.2
260.04	0.1641	718.91	3.684	0.1808	5.1
264.93	0.1980	711.94	4.393	0.1729	4.9
270.02	0.2386	704.57	5.239	0.1643	4.8
275.11	0.2855	697.08	6.205	0.1565	4.6
280.09	0.3377	689.64	7.279	0.1498	4.5
285.08	0.3972	682.04	8.495	0.1428	4.4
290.09	0.4645	674.27	9.869	0.1362	4.3
295.09	0.5398	666.36	11.41	0.1301	4.2
300.08	0.6237	658.29	13.13	0.1243	4.1
305.17	0.7191	649.88	15.09	0.1186	3.9
310.08	0.8208	641.56	17.2	0.1136	3.9
315.07	0.9350	632.88	19.59	0.1085	3.8
320.12	1.062	623.85	22.28	0.1033	3.7
325.08	1.199	614.71	25.21	0.09862	3.6
330.06	1.348	605.23	28.49	0.09447	3.5
334.96	1.509	595.56	32.08	0.09022	3.5
340.11	1.692	584.98	36.29	0.08644	3.4
345.23	1.890	573.98	40.99	0.08147	3.3
350.13	2.095	562.93	46.04	0.07814	3.3

* Calculated with NIST computer program REFPROP Version 9.0 [52].

**Table 4:** Averaged experimental viscosities for saturated liquid R1234yf

Temperature *T*	Pressure* *P*	Saturated Liquid Density* *ρ*_SL_	Saturated Vapor Density* *ρ*_SV_	Viscosity *η*	Combined Standard Uncertainty *u*(*η*)
K	MPa	kg·m^−3^	kg·m^−3^	mPa·s	%
246.54	0.1148	1255.1	6.724	0.2826	5.2
250.01	0.1328	1245.3	7.716	0.2699	5.1
255.01	0.1626	1230.9	9.343	0.2541	4.8
260.04	0.1975	1216.2	11.24	0.2390	4.6
265.00	0.2373	1201.4	13.40	0.2260	4.5
270.00	0.2834	1186.1	15.89	0.2116	4.0
275.01	0.3362	1170.4	18.75	0.1984	3.9
280.01	0.3960	1154.4	22.00	0.1901	3.8
285.00	0.4635	1137.9	25.69	0.1789	3.7
290.00	0.5393	1120.9	29.88	0.1687	3.6
294.98	0.6239	1103.4	34.60	0.1626	3.3
299.99	0.7186	1085.1	39.98	0.1548	3.2
304.99	0.8233	1066.3	46.05	0.1462	3.2
310.00	0.9393	1046.6	52.95	0.1393	3.1
315.00	1.067	1026.1	60.76	0.1304	3.0
320.00	1.207	1004.6	69.65	0.1246	2.9
325.00	1.361	981.81	79.80	0.1162	2.8
329.98	1.528	957.66	91.43	0.1091	2.7
335.01	1.712	931.36	105.0	0.1015	2.6
340.01	1.911	902.77	120.9	0.09432	2.6

* Calculated with NIST computer program REFPROP Version 9.0 [52].

**Table 5:** Averaged experimental viscosities for saturated liquid R1234ze(E)

Temperature *T*	Pressure* *P*	Saturated Liquid Density* *ρ*_SL_	Saturated Vapor Density* *ρ*_SV_	Viscosity *η*	Combined Standard Uncertainty *u*(*η*)
K	MPa	kg·m^−3^	kg·m^−3^	mPa·s	%
246.73	0.07237	1313.4	4.164	0.3735	5.2
250.00	0.08412	1304.7	4.795	0.3569	5.0
255.00	0.1050	1291.2	5.903	0.3321	4.7
260.02	0.1297	1277.5	7.209	0.3078	4.5
265.00	0.1586	1263.6	8.721	0.2899	4.3
269.99	0.1924	1249.4	10.48	0.2730	4.1
275.00	0.2316	1234.8	12.51	0.2570	4.0
280.00	0.2767	1220.0	14.83	0.2414	3.9
285.00	0.3282	1204.8	17.49	0.2284	3.7
290.00	0.3866	1189.2	20.52	0.2135	3.6
294.99	0.4527	1173.3	23.95	0.2022	3.5
300.01	0.5272	1156.8	27.86	0.1906	3.4
305.00	0.6102	1140.0	32.26	0.1805	3.3
310.00	0.7030	1122.6	37.23	0.1705	3.3
315.00	0.8060	1104.6	42.84	0.1608	3.2
320.00	0.9198	1085.9	49.18	0.1523	3.1
325.00	1.045	1066.5	56.35	0.1418	3.1
330.00	1.183	1046.1	64.46	0.1339	3.0
335.00	1.334	1024.5	73.67	0.1250	2.9
340.00	1.499	1001.5	84.18	0.1168	2.7

* Calculated with NIST computer program REFPROP Version 9.0 [52].
